# Challenges in the pathological diagnosis of erythropoietic protoporphyria: a case report

**DOI:** 10.3389/fmed.2025.1664961

**Published:** 2025-09-25

**Authors:** Tingting Yang, Changxian Chen, Chunming Li, Jinjing Wang

**Affiliations:** Department of Pathology, Affiliated Hospital of Zunyi Medical University, Zunyi, China

**Keywords:** erythropoietic protoporphyria, FECH, pathological diagnosis, liver damage, Gilbert’s syndrome

## Abstract

**Background:**

Erythropoietic protoporphyria (EPP) is a rare autosomal recessive disorder caused by mutations in the FECH gene, leading to ferrochelatase deficiency and the accumulation of protoporphyrin in various organs. EPP patients with liver damage have atypical histopathological manifestations, which pose challenges for pathological diagnosis.

**Content:**

We report a 29-year-old male with recurrent abdominal pain and scleral jaundice. Initial liver dysfunction suggested autoimmune hepatitis, but liver biopsy revealed dense brownish granular deposits with red birefringence under polarized light, characteristic of EPP. Genetic testing identified a new mutation site (c.804 + 1del), which may be related to the disease. Additionally, the patient also has Gilbert’s syndrome.

**Conclusion:**

This case highlights the diagnostic challenges of EPP and the importance of integrating clinical history, histopathology, and genetic testing. Polarized light microscopy is crucial for identifying the characteristic features of EPP. Early genetic testing can guide timely diagnosis and treatment, contributing to the understanding of this rare condition.

## Introduction

Porphyrias are a group of hereditary metabolic disorders caused by defects in enzymes involved in the heme biosynthesis pathway. EPP is an autosomal recessive disorder resulting from the loss of ferrochelatase activity, which disrupts heme biosynthesis and leads to the accumulation of protoporphyrin in various tissues, including the skin, blood, and liver (1). This accumulation causes the characteristic clinical manifestations of the disease. EPP is classified as a rare condition, with incidence rates reported in international studies ranging from 2 to 5 cases per 1,000,000 individuals (2). Clinically, EPP mainly manifests as increased skin sensitivity, with a small portion affecting the liver. Macroscopic examination of the liver in EPP patients typically reveals a dark appearance, while microscopic analysis shows cholestatic changes and birefringent deposits, which form a characteristic Maltese cross pattern under polarized light microscopy. This report describes a case of an EPP patient admitted for abnormal liver function, who was diagnosed after a detailed medical history review, liver biopsy, and whole-exome sequencing. Additionally, a novel mutation, c.804 + 1del, was identified in the FECH gene, which may be associated with the disease.

### Admission history

A 29-year-old man was hospitalized multiple times in April 2022, December 2023, and September 2024 due to recurrent abdominal pain and scleral jaundice. Upon the first admission, abnormal liver function was observed, with laboratory results as follows: Alanine Aminotransferase (ALT) 412 U/L (Reference Range: 0–40 U/L), Aspartate Aminotransferase (AST) 201 U/L (Reference Range: 0–40 U/L), Total Bilirubin 221.1 μmol/L (Reference Range: 0–6.8 μmol/L), Direct Bilirubin 118 μmol/L (Reference Range: 3.4–17.1 μmol/L), and Albumin 41.2 g/L (Reference Range: 35–55 g/L). The autoimmune hepatitis antibody test results were as follows: antinuclear antibody (ANA) (1:40) positive (nucleolar type), antinuclear antibody (ANA) (1:80) weakly positive (nucleolar type), antinuclear antibody (ANA) (1:100) weakly positive (nucleolar type), smooth muscle antibody negative, and mitochondrial antibody negative. The patient’s past medical history was unremarkable. He reported a family history of “photosensitivity,” although no similar cases were noted in his family. On admission, the patient exhibited severe jaundice of the skin and sclera, a soft abdomen with localized tenderness, but no rebound tenderness.

### Imaging examinations

**Figure 1:**
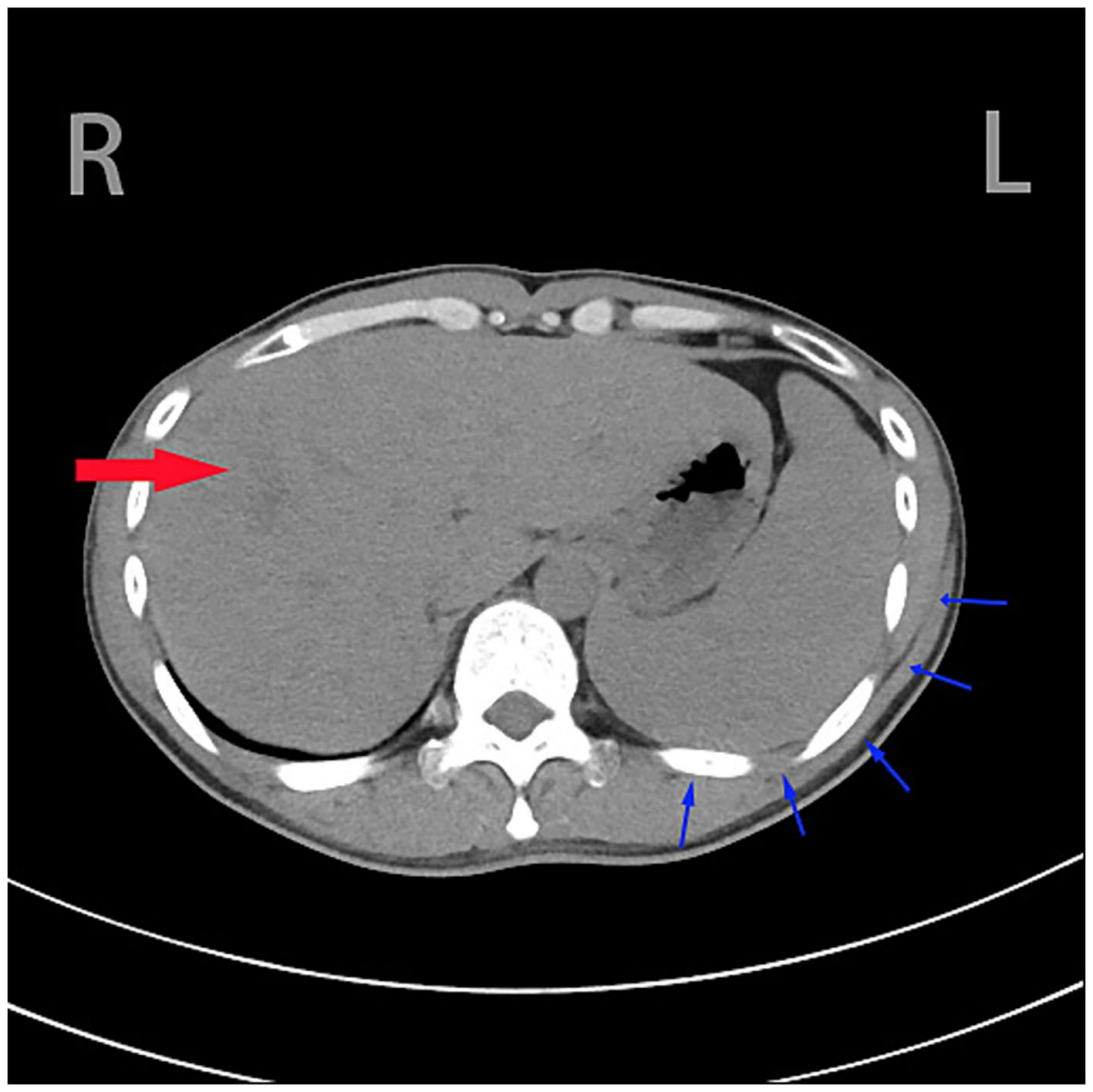
Heterogeneous hepatic parenchymal density is observed (denoted by red arrow), accompanied by splenomegaly with the splenic outer margin extending beyond five rib units (indicated by blue arrow).

Routine abdominal ultrasound revealed hypoechoic areas at the gallbladder fundus, suggesting bile stasis and sludge formation. Abdominal CTA showed portal hypertension, lower esophageal varices, splenomegaly, uneven liver parenchymal density, and mild dilation of the intrahepatic bile ducts (Figure 1).

### Laboratory examinations

Upon admission, the patient was initially diagnosed with unexplained liver dysfunction. A systematic examination was conducted to exclude common causes of liver damage. The patient denied a history of alcohol consumption. Serological markers for Hepatitis B virus, HIV antibodies, Hepatitis C virus antibodies, Hepatitis A virus antibodies, and Hepatitis E virus antibodies were all negative, ruling out viral hepatitis. Alpha-fetoprotein (AFP) testing revealed no abnormalities, ruling out liver cancer. Autoantibody tests (including GBM, PR3, MPO, etc.) excluded liver injury associated with systemic or connective tissue diseases. Autoantibodies for autoimmune hepatitis were partially positive, autoimmune hepatitis cannot be ruled out (3). Given this situation, liver biopsy was performed for pathological examination.

### Pathological findings

#### Gross examination

A black liver tissue sample, approximately 7 mm in length and 1 mm in diameter, was obtained through biopsy.

#### Microscopic examination

**Figure 2:**
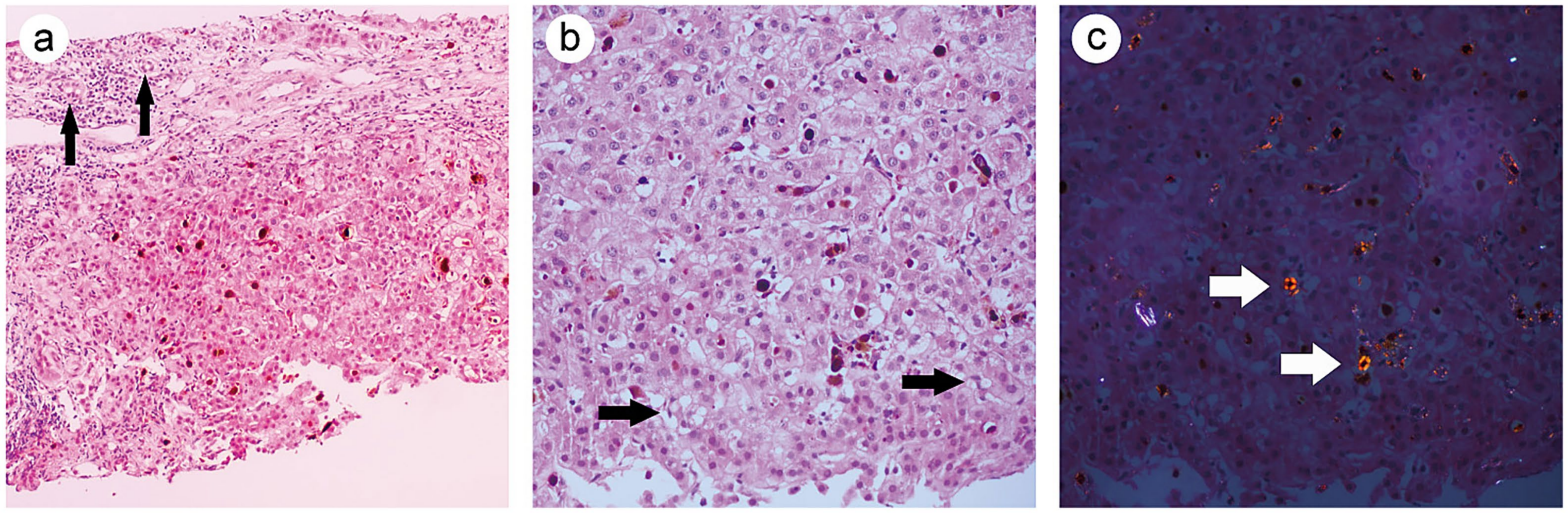
**(a)** Nodular hepatocellular hyperplasia with surrounding fibrous septa. Bile duct proliferation is observed in the portal area (denoted by black arrow) (HE, 20×). **(b)** Hepatocellular steatosis (denoted by black arrow), dense brown granular deposits are visible within hepatic sinusoids and hepatocytes (HE, 40×). **(c)** Polarized light microscopy shows red birefringence of the granular material, and the characteristic Maltese cross structure is visible (indicated by white arrowheads) (HE, 40×).

**Figure 3:**
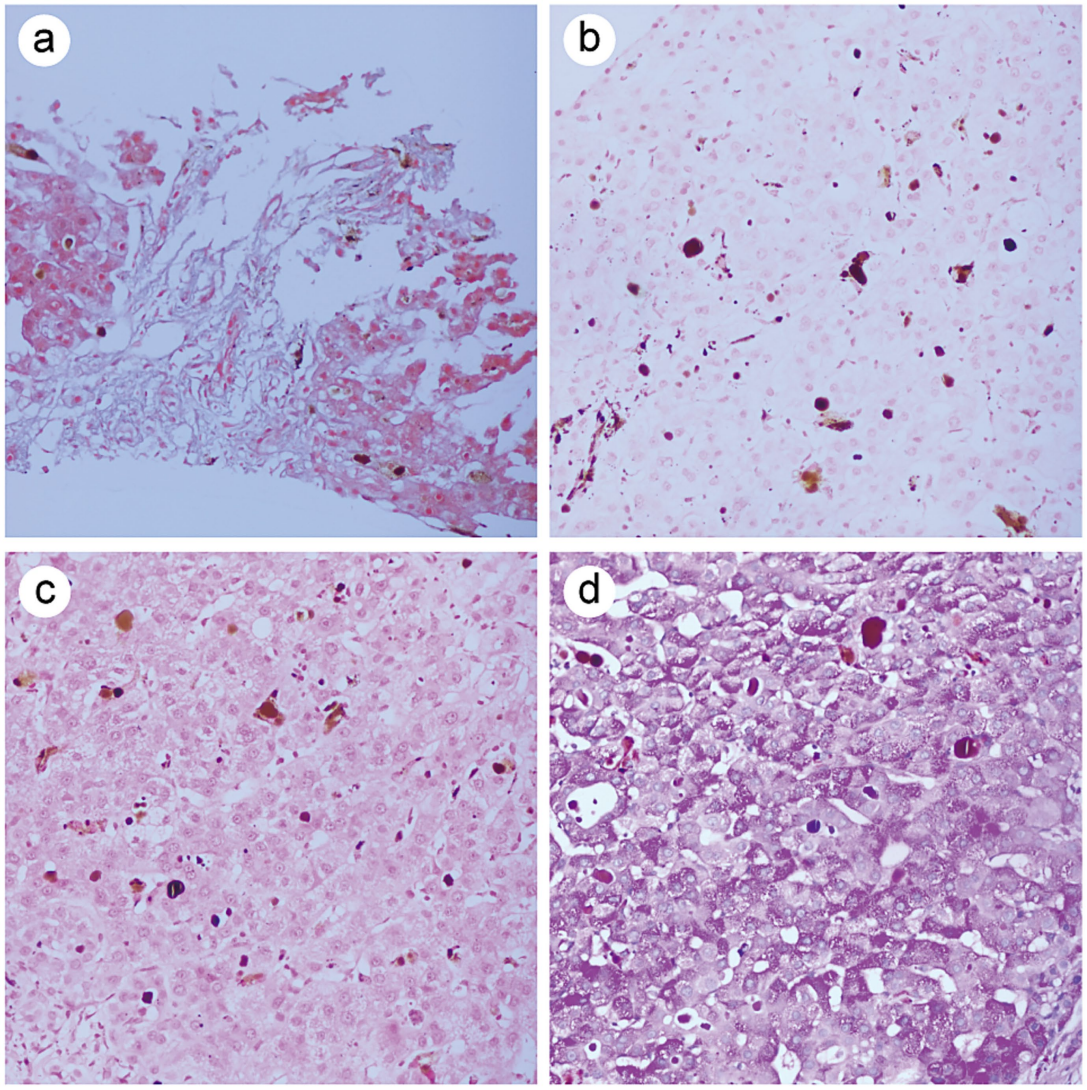
Special staining results. **(a)** The positive expression of Masson staining. **(b)** The negative expression of iron staining. **(c)** The negative expression of copper staining. **(d)** The negative expression of glycogen staining.

Destruction of the liver lobular architecture, with proliferation of fibrous tissue in the portal areas, leading to the formation of hepatocyte clusters. Infiltration of inflammatory cells is observed in the stroma, along with bile duct hyperplasia, mild hydropic degeneration, and mild fatty change in hepatocytes (Figure 2a). Dense brownish granular deposits were observed in the hepatic sinusoids and hepatocytes (Figure 2b). Under polarized light microscopy, the granular deposits exhibited red birefringence, with a characteristic Maltese cross pattern (Figure 2c). Immunohistochemical staining results showed negative CD138, CD4, CD8, IgG, IgG4 and IgM, while small bile ducts were positive for CK7 and CK19 (Supplementary Figures S1–S8). Special staining results showed positive Masson staining, with negative results for iron staining, copper staining, and glycogen staining (Figures 3a–d). The final pathological diagnosis was EPP complicated by cirrhosis.

### Genetic Testing

**Figure 4:**
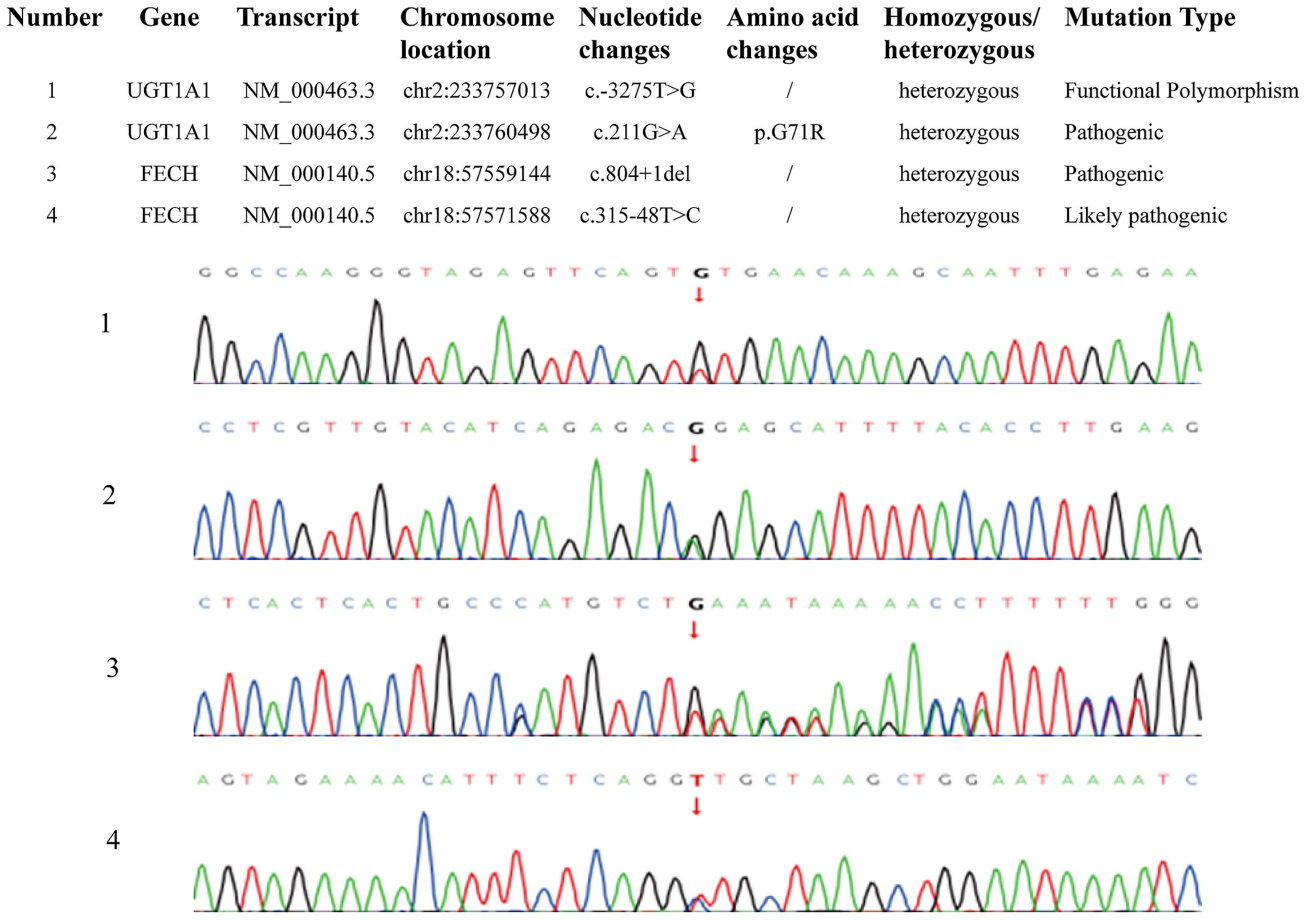
Genetic testing results.

Subsequently, genetic testing for porphyria-related genes was recommended. This genetic testing employed whole-exome sequencing (WES) utilizing a target capture-based high-throughput sequencing methodology, with reference to the GRCh38 (hg38) human reference genome assembly. The test results revealed two heterozygous variants in the patient’s FECH gene: c.804 + 1del (4, 5) and c.315-48 T > C (6, 7). Additionally, a heterozygous single nucleotide substitution, c.-3275 T > G, was detected in the upstream regulatory region of the patient’s UGT1A1 gene, and a heterozygous missense mutation, c.211G > A:p. G71R, was identified in the coding region (8–10) (Figure 4). The final genetic diagnosis was FECH gene abnormalities leading to autosomal recessive EPP, combined with UGT1A1 gene abnormalities resulting in autosomal recessive Gilbert syndrome (11).

### Treatment and prognosis

The patient was advised for photoprotection and initiated on oral methylprednisolone tablets (24 mg once daily) and thalidomide (25 mg three times daily), supplemented with polyene phosphatidylcholine for hepatoprotection. Following a course of supportive therapy, the patient’s condition stabilized with resolution of jaundice. Upon discharge, the patient was instructed to maintain strict photoprotection and consider oral *β*-carotene supplementation if photosensitivity manifestations occur. Concurrent avoidance of alcohol consumption was emphasized. Regular monitoring of liver function and hepatitis B-related serological markers was arranged, with ongoing follow-up after discharge.

## Discussion

EPP is an autosomal recessive genetic disorder caused by a deficiency of ferrochelatase activity in the heme biosynthesis pathway, leading to the accumulation of protoporphyrin in tissues such as the skin, blood, and liver, thereby triggering associated clinical symptoms. The clinical presentation of EPP is primarily characterized by heightened skin sensitivity, with a minority of patients developing liver damage. The liver of EPP patients appears dark upon macroscopic examination, and under microscopy, dense brown-red protoporphyrin polymer crystals can be observed within the bile canaliculi, hepatocytes, and Kupffer cells. These crystals exhibit birefringence under polarized light microscopy, showing a “starry sky” appearance, with some displaying the characteristic Maltese cross pattern (1). Additionally, varying degrees of hepatocyte degeneration and necrosis are observed, with more severe cases showing different levels of fibrosis and cirrhosis.

The patient in this case was admitted multiple times, presenting with abdominal pain and scleral jaundice. Laboratory tests indicated abnormal liver function and partial positivity for autoimmune hepatitis antibodies. Autoimmune hepatitis was initially considered clinically, and a liver biopsy was sent to the pathology department. Hematoxylin and eosin (HE) staining revealed destruction of the hepatic lobular architecture, proliferation of fibrous tissue in the portal areas, with lobular division leading to the formation of hepatocyte clusters. Cholestatic changes are observed in both hepatocytes and hepatic sinusoids. After repeated careful slide examination by the pathologist, immunohistochemistry and special staining were performed. The results showed positive Masson’s trichrome staining, with small bile ducts expressing CK7 and CK19, consistent with cirrhosis. CD138, CD4, CD8, IgG, IgG4, and IgM were negative, ruling out autoimmune hepatitis. Special staining results showed negative staining for iron, copper, and glycogen, further excluding hemochromatosis (12), Wilson’s disease (13), and glycogen storage diseases (14). At this point, the cause of the patient’s condition remained unclear. Microscopic examination revealed morphological abnormalities in bile plugs within hepatocytes and liver sinusoids. These brownish substances, resembling bile plugs, had a deeper color and denser structure, whereas typical bile plugs appear homogeneous, lightly stained, and lack granular material. The tissue biopsy report notes the presence of an abnormal black coloration in the liver specimen. Considering the patient’s relatively young age, the pathologist suspected porphyria. After repeated history taking, the patient informed the clinical team of a family history of “skin photosensitivity,” suggesting the possibility of porphyria. The pathologist further observed these brownish particles under polarized light microscopy, where they showed red birefringence. Some particles exhibited the characteristic Maltese cross pattern, consistent with the pathological features of porphyria.

Given the complexity of porphyria classification, genetic testing was recommended. The results revealed abnormalities in the FECH gene, with two heterozygous variants: c.804 + 1del and c.315-48 T > C. EPP is primarily caused by FECH gene mutations. In this case, the patient has autosomal recessive inheritance, and the clinical features of the disease caused by these mutations align with the patient’s symptoms, including the family history of “skin photosensitivity,” abdominal pain, and scleral jaundice. Notably, whole-exome sequencing also revealed a canonical splice-site mutation in the FECH gene (NM_000140.5: c.804 + 1G > C). This variant involves a substitution of guanine (G) to cytosine (C) at the +1 position of intron 804 of the FECH gene, which is a highly conserved core nucleotide of the donor splice site. Consequently, this change is expected to disrupt normal RNA splicing, leading to a loss of function (LOF) via aberrant transcript processing. This variant has not been reported in the gnomAD population database. In accordance with the guidelines established by the American College of Medical Genetics and Genomics (ACMG) and the Clinical Genome Resource (ClinGen), this variant is classified as pathogenic and is predicted to adversely affect the structure and function of the FECH protein. Additionally, the patient was found to have mutations in the UGT1A1 gene, including a heterozygous single nucleotide substitution variant in the upstream regulatory region (c.-3275 T > G) and a heterozygous missense mutation in the coding region (c.211G > A:p. G71R). The UGT1A1 gene mutation leads to autosomal recessive Gilbert syndrome. Gilbert syndrome, also known as congenital non-hemolytic jaundice or hereditary unconjugated hyperbilirubinemia, is a disorder of bilirubin metabolism that typically presents with subtle onset and usually without obvious symptoms. The clinical hallmark is elevated unconjugated bilirubin. The disease does not damage hepatocytes or bile duct cells, so liver enzymes (ALT, AST) and biliary enzymes (ALP, *γ*-GGT) are typically normal. In this case, the patient’s direct bilirubin (118 μmol/L) and total bilirubin (221.1 μmol/L) were both elevated, suggesting an increase in indirect bilirubin, which is consistent with the clinical features of Gilbert’s syndrome. However, the patient also had elevated liver enzymes (ALT 412 U/L, AST 201 U/L), which cannot be excluded as a result of EPP with concomitant cirrhosis. Due to financial constraints, the patient did not undergo family genetic pedigree testing.

In summary, the clinical presentation of this patient was atypical, with no obvious skin lesions. Due to limitations in testing conditions, quantitative measurements of blood, urine, and fecal protoporphyrin were not performed, which significantly complicated the diagnosis. Ultimately, through close collaboration between the pathologist and the clinical team, EPP was diagnosed, confirmed by genetic testing. EPP is a rare genetic metabolic disorder that is prone to misdiagnosis, necessitating pathologists to have extensive diagnostic experience. In routine pathological diagnosis, when dense brown particles are observed within liver sinusoids or hepatocytes, in addition to considering bile plug formation, other metabolic liver diseases, such as Wilson’s disease, glycogen storage diseases, and hemochromatosis, should also be considered, which can be differentiated through special staining. Diagnosing EPP requires pathologists to make a comprehensive diagnosis based on the patient’s history, clinical presentation, and laboratory test results. When EPP is suspected, polarized light microscopy should be employed to observe whether the characteristic pathological features are present. Early genetic testing should be conducted, and active treatment should be initiated promptly after diagnosis to improve the patient’s prognosis.

## Data Availability

The original contributions presented in the study are included in the article/Supplementary material, further inquiries can be directed to the corresponding authors.
