# Supplementary figures and images for: Challenges in the pathological diagnosis of erythropoietic protoporphyria: a case report
Source: Front Med (Lausanne). 2025 Sep 25;12:1664961. doi: 10.3389/fmed.2025.1664961 (PMC12507767; doi:10.3389/fmed.2025.1664961)

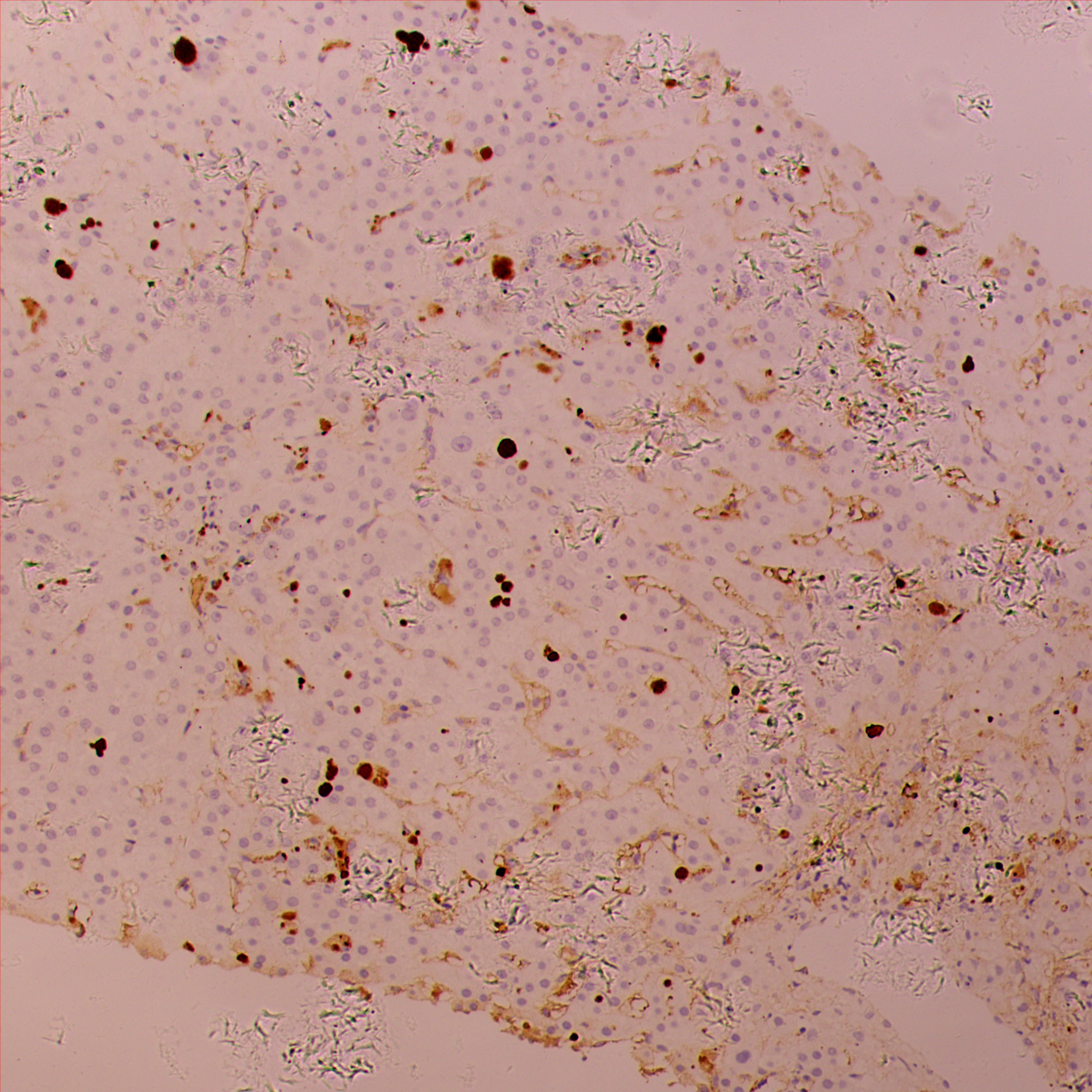

Supplement: Supplementary file 1 [file Image_1.PNG]

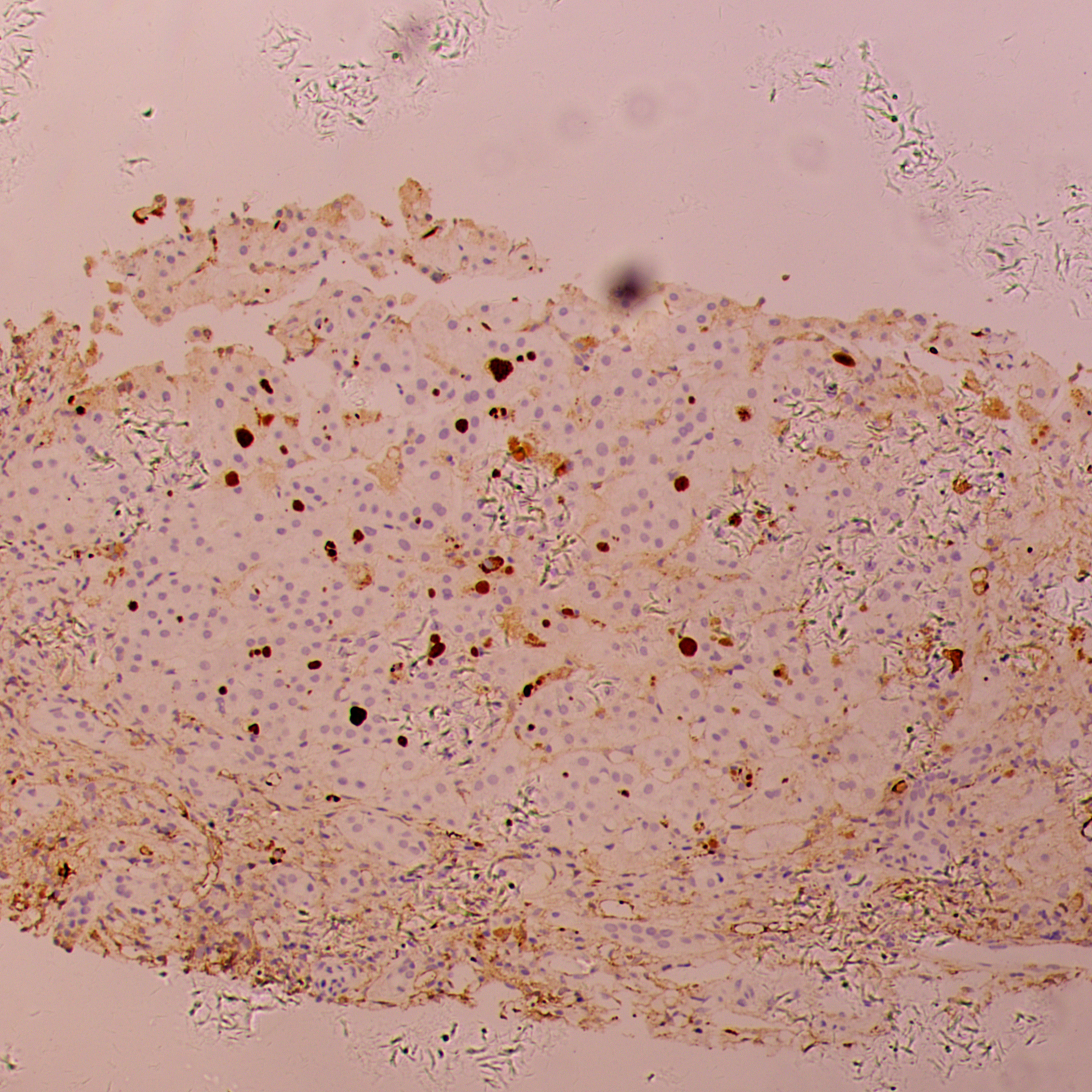

Supplement: Supplementary file 2 [file Image_2.PNG]

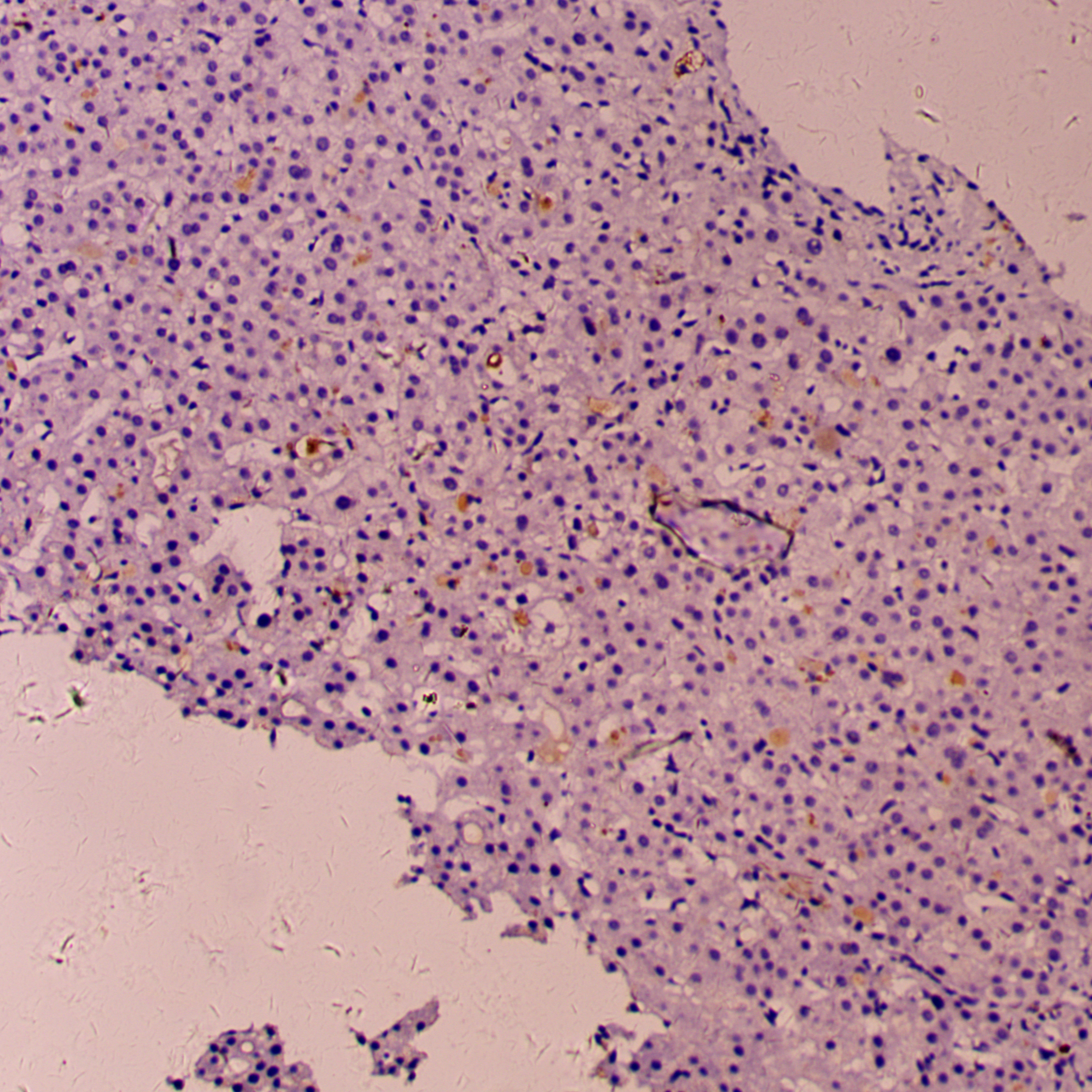

Supplement: Supplementary file 3 [file Image_3.PNG]

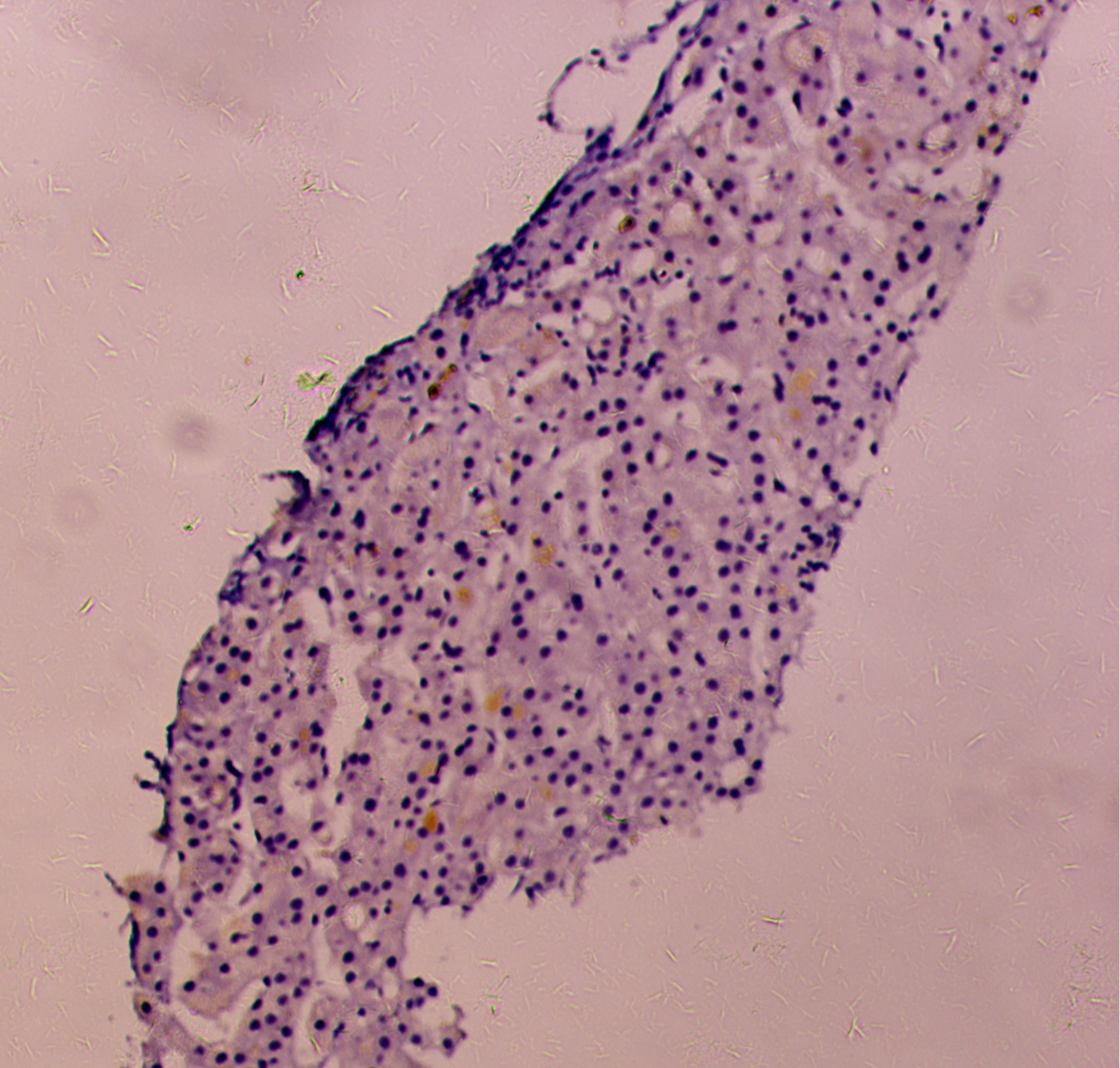

Supplement: Supplementary file 4 [file Image_4.PNG]

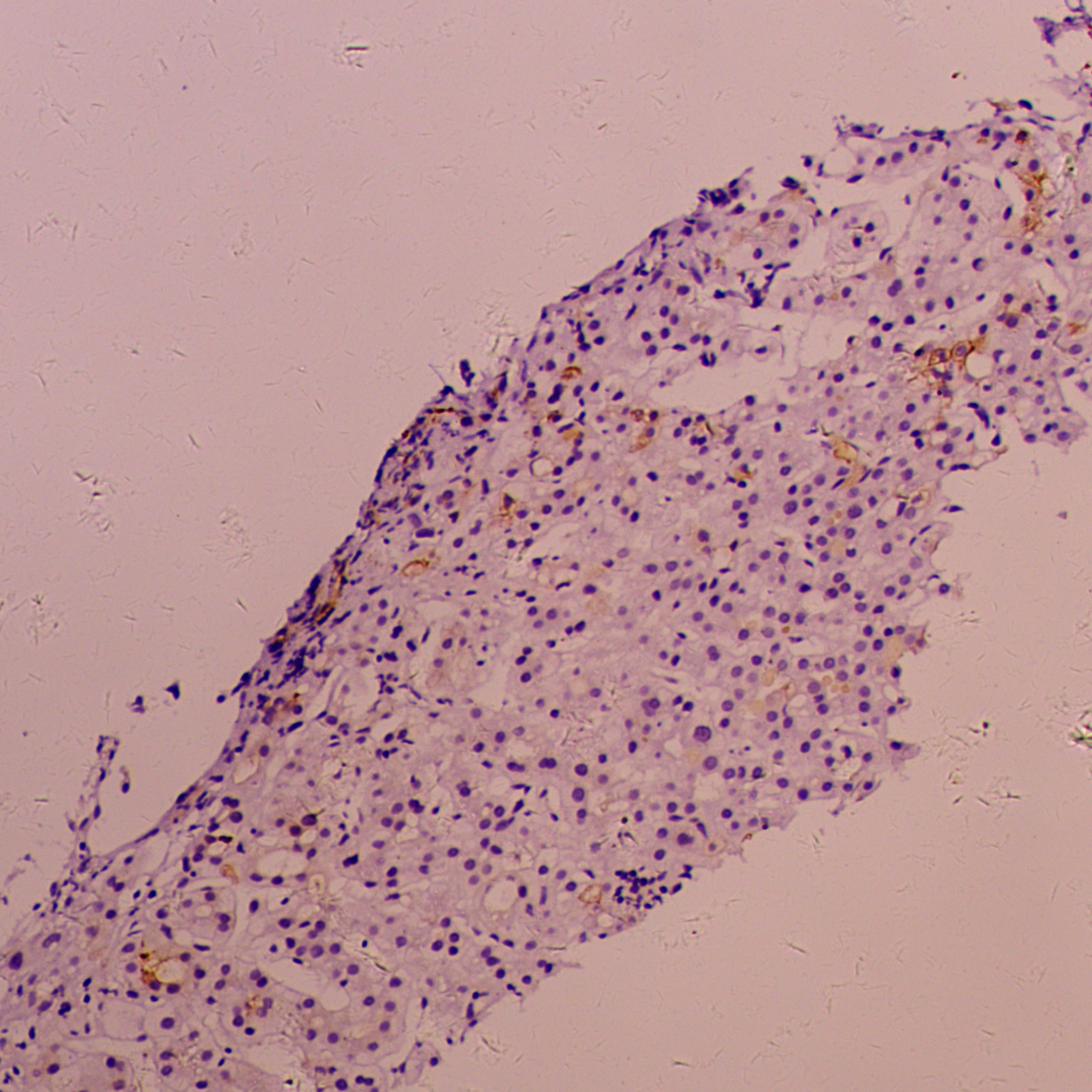

Supplement: Supplementary file 5 [file Image_5.PNG]

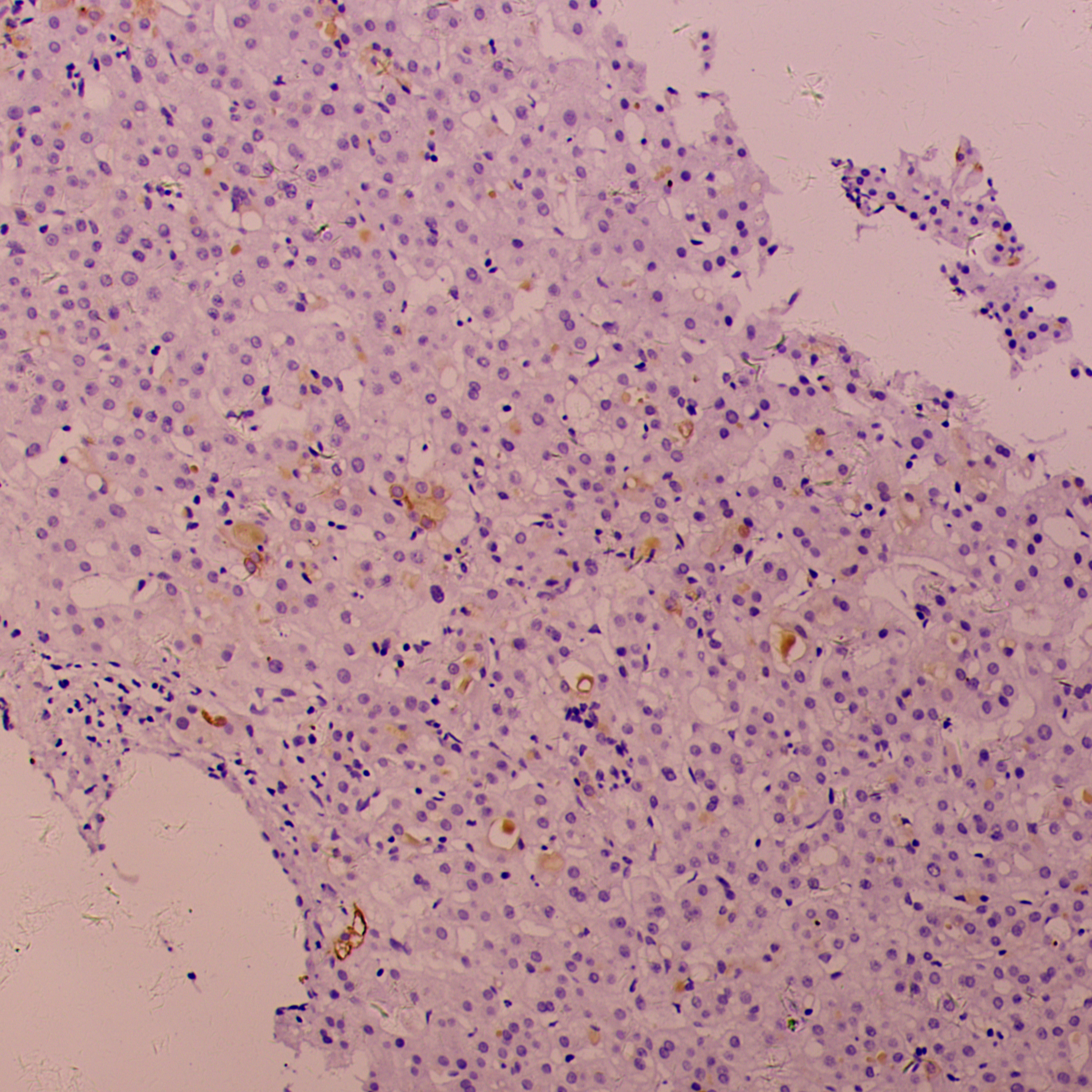

Supplement: Supplementary file 6 [file Image_6.PNG]

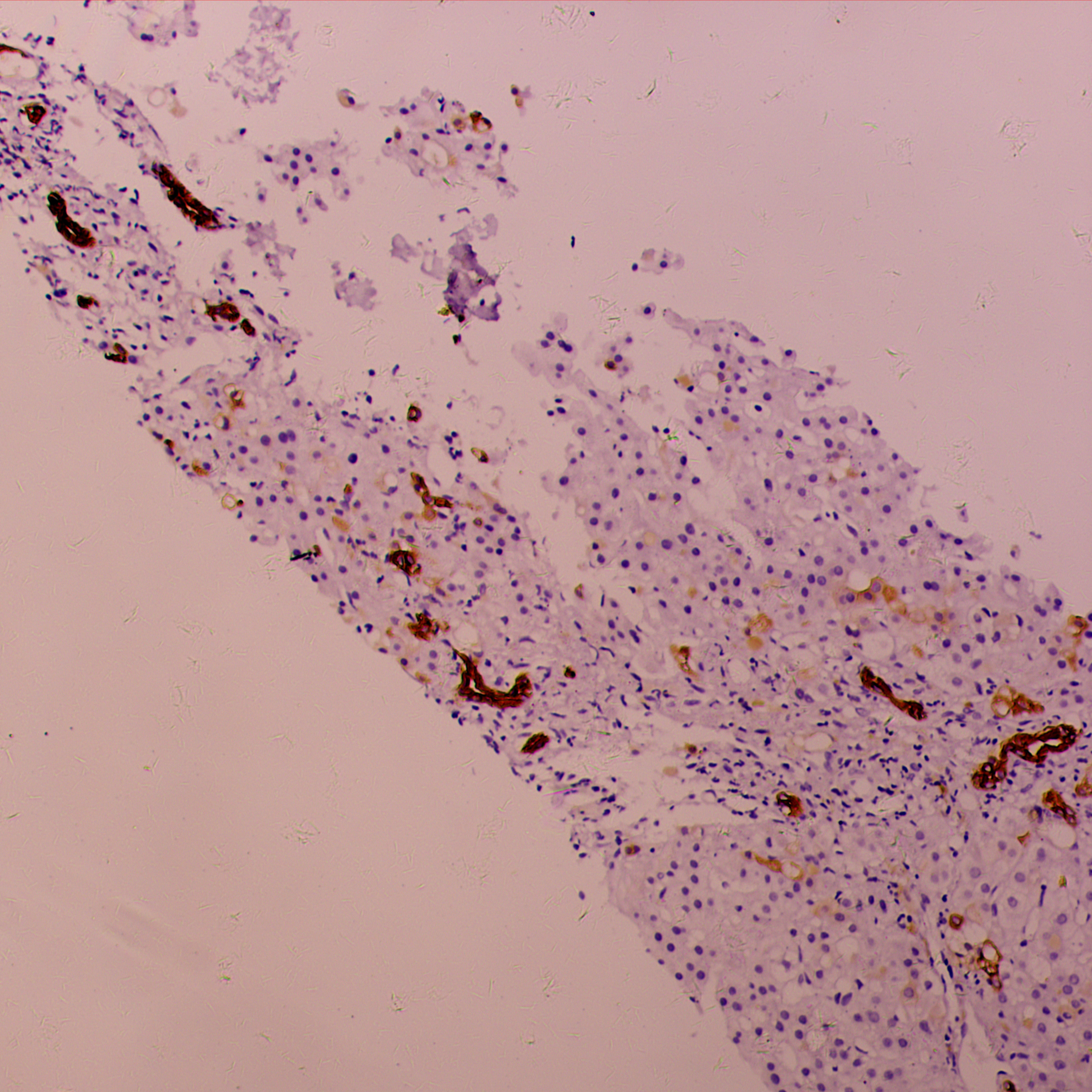

Supplement: Supplementary file 7 [file Image_7.JPEG]

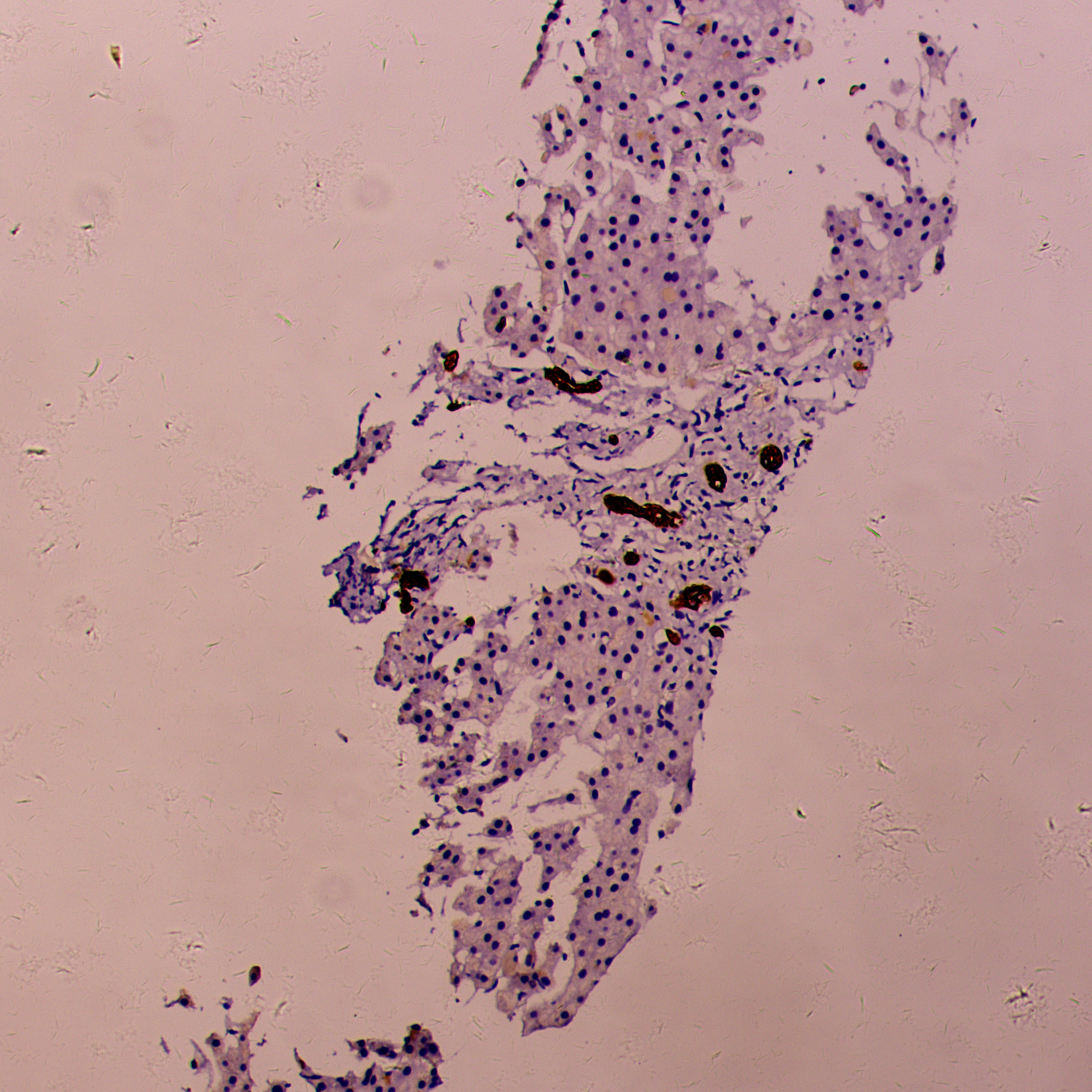

Supplement: Supplementary file 8 [file Image_8.JPEG]
